# Diagnostics Using Non-Invasive Technologies in Dermatological Oncology

**DOI:** 10.3390/cancers14235886

**Published:** 2022-11-29

**Authors:** Simone Soglia, Javiera Pérez-Anker, Nelson Lobos Guede, Priscila Giavedoni, Susana Puig, Josep Malvehy

**Affiliations:** 1 Melanoma Unit, Dermatology Department, Hospital Clínic de Barcelona, IDIBAPS, Universitat de Barcelona, 08001 Barcelona, Spain; 2 Department of Dermatology, University of Brescia, 25121 Brescia, Italy

**Keywords:** diagnostics, skin cancer, artificial intelligence, imaging, imaging techniques, RCM, OCT, LC-OCT, total body photography (TBP), high frequency ultrasound

## Abstract

**Simple Summary:**

Skin tumors are appearing with increasing frequency worldwide. To face this health problem, new technologies and devices have been developed in recent years, allowing the acquisition of information about healthy and neoplastic skin, unavailable until a few years ago. Dermatologists need, therefore, continuous training and updating to ensure adequate patient diagnosis and treatment. This article aims to review the currently available technologies for the screening, diagnosis, mapping and monitoring in dermatological oncology highlighting their applications, limitations and possible future perspectives.

**Abstract:**

The growing incidence of skin cancer, with its associated mortality and morbidity, has in recent years led to the developing of new non-invasive technologies, which allow an earlier and more accurate diagnosis. Some of these, such as digital photography, 2D and 3D total-body photography and dermoscopy are now widely used and others, such as reflectance confocal microscopy and optical coherence tomography, are limited to a few academic and referral skin cancer centers because of their cost or the long training period required. Health care professionals involved in the treatment of patients with skin cancer need to know the implications and benefits of new non-invasive technologies for dermatological oncology. In this article we review the characteristics and usability of the main diagnostic imaging methods available today.

## 1. Introduction

Skin cancer constitutes the most common malignancy in mankind [1]. Both melanoma (MM) and non-melanoma skin cancers (NMSC) show a progressively increasing incidence worldwide, especially in the caucasian population [2]. This rising incidence rate represents a growing health problem, due both to tumor-associated morbidity and mortality and to the economic burden related to monitoring and treatment. In the last few years, with advances in technologies, new in vivo and ex vivo diagnostic techniques have been developed in an attempt to obtain an ever more precise and early diagnosis [3,4].

Digital photography, total-body photography (TBP) (2D or 3D), dermoscopy, reflectance confocal microscopy (RCM), optical coherence tomography (OCT), line-field confocal optical coherence microscopy (LC-OCT) and high frequency ultrasound (HFUS) are the most commonly used imaging methods for the diagnosis and monitoring of skin cancers. Increasing evidence supporting the efficacy of some of these modern methods in the diagnosis of skin cancer has led to their being incorporated into the recommendations of international guidelines [5,6].

This article reviews the available non-invasive technologies where applied to screening, diagnosis and monitoring in dermatological oncology, highlighting their applications, limitations, and possible future perspectives (Table 1).

**Figure 1:**
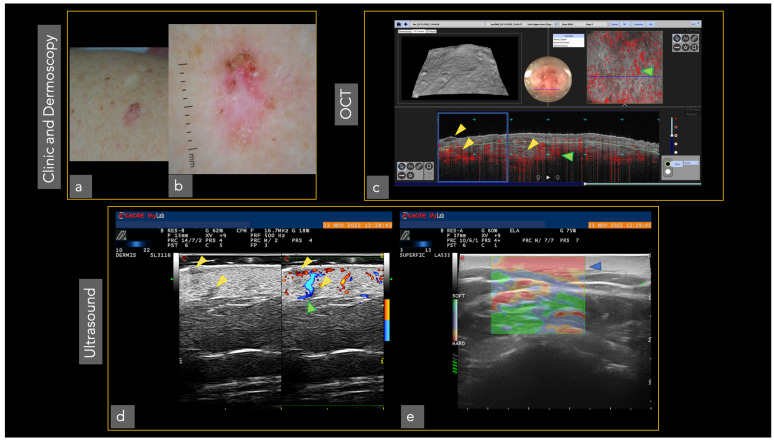
Superficial and infiltrating basal cell carcinoma in the left arm was studied with different techniques. Tumor presence (yellow triangles). The vascular pattern is represented with green triangles: (**a**) Clinical image; (**b**) Dermoscopy; (**c**) OCT showing superficial and infiltrating basal cell carcinoma (blue square). The tumor surface is perceived (superior left). Dermoscopy area scanned (center). The vascular pattern can also be observed in 3 D (superior right); (**d**) The same tumoral islands are observed in ultrasound (left). The addition of the Doppler image reveals the complete tumor invasion (right); (**e**) The elastography shows the denser area corresponding to the tumor (blue triangle). Ultrasound image courtesy of Dr. Priscila Giavedoni.

**Figure 2:**
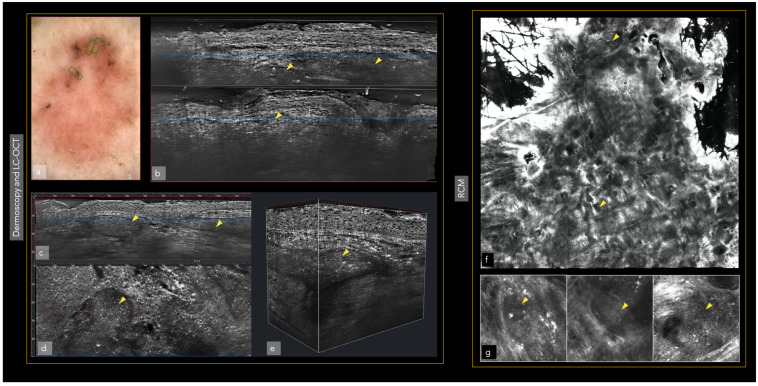
The same superficial and infiltrating basal cell carcinoma in the left arm also studied with LC-OCT and RCM. Tumor presence (yellow triangles). Blue and red lines are the same area represented in the vertical (blue) and horizontal (red) view of LC-OCT: (**a**) Dermoscopy; (**b**) Vertical view of LC-OCT; (**c**) Vertical view of the tumor islands in LC-OCT; (**d**) The same area observed in the horizontal view of LC-OCT; (**e**) 3D view at the same level; (**f**) RCM revealing superficial and infiltrating basal cell carcinoma; (**g**) RCM tumoral details of the superficial component of the basal cell carcinoma.

Different imaging techniques are represented in Figure 1 and Figure 2, in which the same superficial and infiltrating basal cell carcinoma has been studied with different in vivo techniques. 

## 2. Techniques

### 2.1. Digital Photography and Total Body Photography (TBP)

The skin is the only organ directly accessible from outside the body, so it is not surprising that dermatologists and other professionals involved in skin diseases were among the first to employ digital photography in their discipline [68,69,70,71,72,73,74].

Digital photography is a useful low-cost tool which can assist the clinician in maintaining accurate records, educating patients, and providing medico-legal documentation. Moreover, it allows an objective evaluation of the patient’s lesions over time, providing both qualitative and quantitative information. It is, therefore, an essential tool in skin tumor examination and in pre-surgical planning. However, a lack of integration of clinical photography into the electronic health records (EHR) of patients in many dermatological clinics represents a barrier to the universal use of this practice.

TBP permits the acquisition of images of the entire body in one single action. Furthermore, new devices use high resolution cameras and polarized light to enhance the quality of the images. 

**Figure 3:**
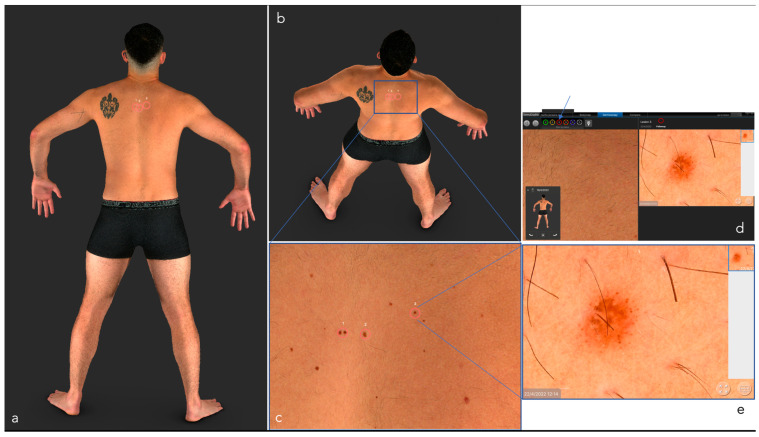
Total body photography. VECTRA WD 360. CANFIELD ^®^3-Dimensional.Each selected lesion receives a number. Lesion “3” (blue arrow) is amplified as an example of the magnification: (**a**) 3D total body reconstruction; (**b**) Selected area to be analyzed; (**c**) Melanocytic nevus in follow up; (**d**) Macroscopic view of the selected nevus in the screen. Red circle shows the nevus in follow up; (**e**) Image magnification of the selected nevus. Details in blue squares.

**Figure 4:**
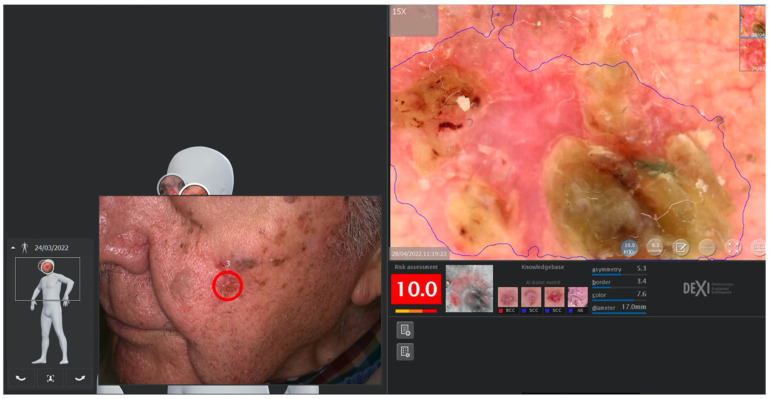
2-Dimensional photography. DermagraPhix^®^. The selected area also includes a dermatoscopic view, with an artificial intelligence score.

This technology can provide a 2-D or 3-D reconstruction of the entire skin surface making it possible to follow up patients with a high number of atypical melanocytic lesions in a relatively short time and to increase the accuracy of the early diagnosis of melanoma [8,9,10,11] (Figure 3 and Figure 4). 

Unfortunately, new TBP technologies still have some limitations. Firstly, there is a lack of standards in acquisition (image format and color, resolution, positioning) and interoperability between different systems. Secondly, TBP technologies have an elevated cost [7] and require a large amount of storage space. The Dermatology DICOM Group aims to report on standards of TBP in a dedicated supplement in 2023 which will facilitate interoperability between different products. 

Finally, clinical photography and dermoscopy in combination have been implemented in teledermatology services and have an important role in dermatological consultations particularly where related to skin cancer. [12]. 

### 2.2. Dermoscopy

In direct naked-eye examination, light is reflected, dispersed or absorbed by the stratum corneum as a function of its refraction index and its optical density, making it impossible to view deeper underlying structures [13,75]. Dermoscopy, using a hand-held magnification device following the application of a liquid at the skin-device interface (reducing light reflection) or using cross-polarized instruments allows the visualization of skin lesions located in the epidermis and upper dermis not seen with the naked eye.

**Figure 5:**
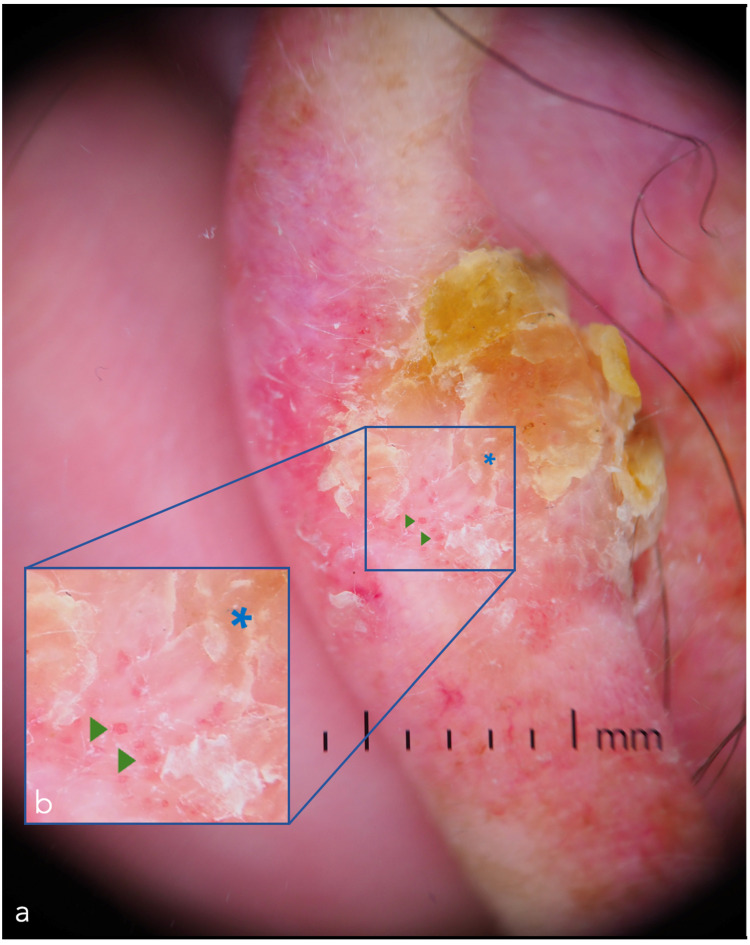
Dermoscopic image of a squamous cell carcinoma of the helix. The presence of yellow-white, amorphous areas of keratin (blue asterisk) and hairpin vessels (green triangles) help the clinician in the diagnosis of this squamous cell carcinoma: (**a**) Dermoscopic image; (**b**) Magnification of the vascular component.

**Figure 6:**
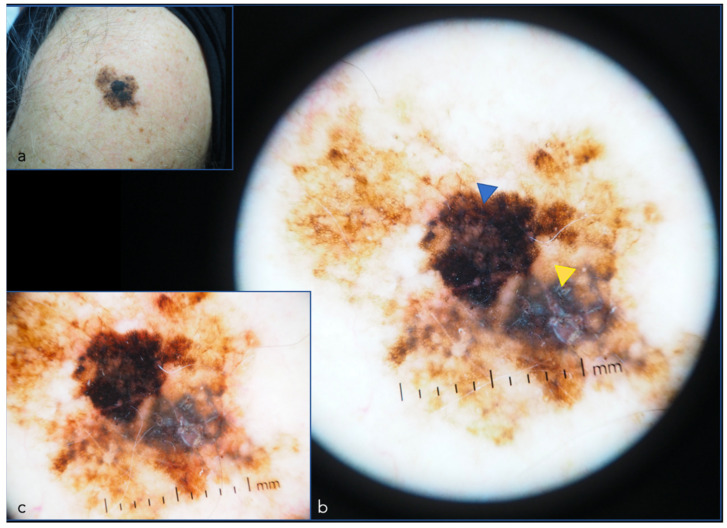
Melanoma in situ (left arm): (**a**) Clinic picture; (**b**) Dermoscopic image. Blue triangle: black structureless area. Yellow triangle: gray structureless area; (**c**) Dermoscopic detail of structureless areas.

**Figure 7:**
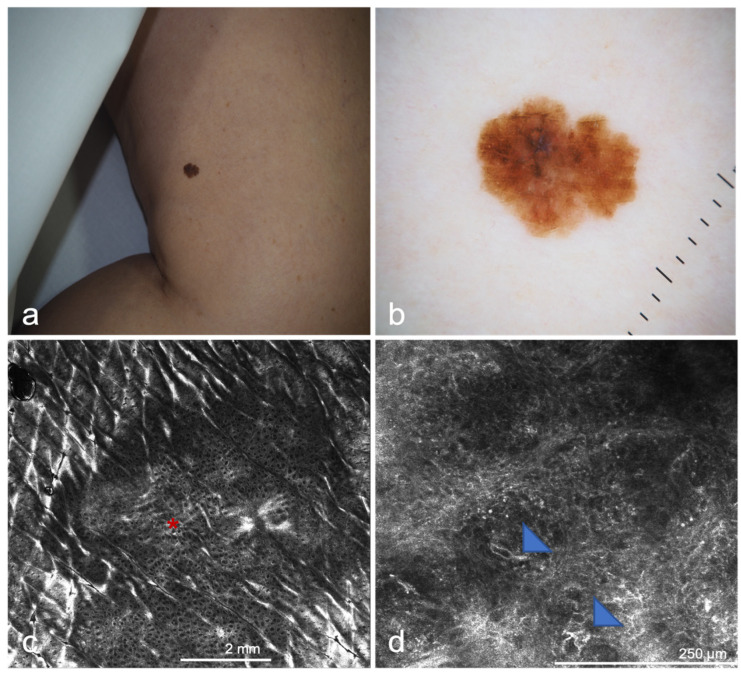
Melanoma in situ on the thigh of a 65-year-old woman: (**a**) Asymmetric, polychrome pigmented lesion with irregular borders; (**b**) Dermoscopic image. Some melanomas show very subtle dermoscopic changes, such as focal color alterations; (**c**) Mosaic of in vivo confocal image. The image was taken at the level of the dermoepidermal junction/upper dermis and shows a ringed pattern focally disrupted (red asterisk); (**d**) Confocal image of the same lesion at the level of the basal layer of the epidermis. The presence of large, bright, dendritic cells with evident nucleus (blue triangles) makes the excision of the lesion necessary.

**Figure 8:**
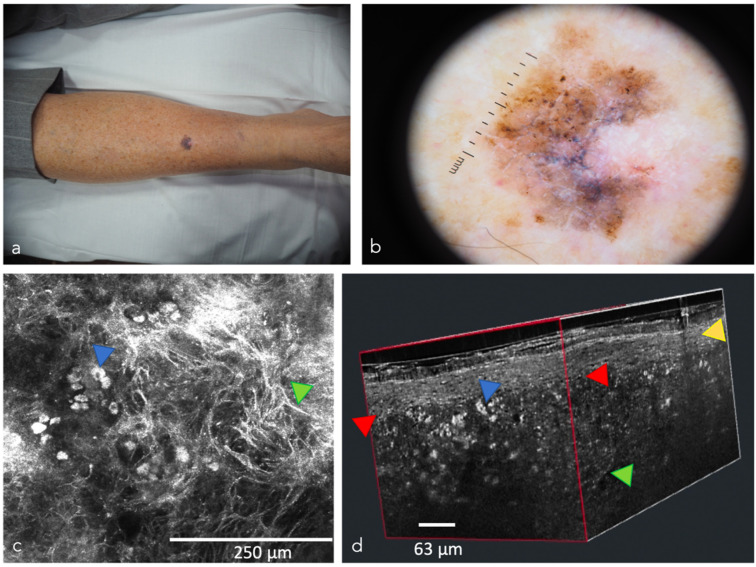
Melanoma in three different imaging techniques: (**a**) Clinical picture; (**b**) Dermoscopic image; (**c**) Reflectance confocal microscopy: cellular atypia (blue arrow) and dendritic cells (green arrow); (**d**) 3D block of LC-OCT: irregular epidermis (yellow arrow), disruption of the dermoepidermal junction (red arrow), atypical nests with atypical (blue arrow) and dendritic cells (green arrow).

Dermoscopy either analog or digital, represents a cornerstone in dermatological diagnostics. It is frequently used for the diagnosis and monitoring of both pigmented and non-pigmented lesions, MM and NMSC helping to achieve an earlier diagnosis (Figure 3) [14]. In addition, it decreases the benign-to-malignant biopsy ratio and allows the diagnosis of thinner MM compared with naked eye examination (Figure 1, Figure 2, Figure 4, Figure 5, Figure 6, Figure 7 and Figure 8) [15,16,17,76]. 

Several diagnostic algorithms have been developed, such as the ABCD rule, the Menzies method, the seven and three-point checklist and the CASH algorithm to improve the sensitivity and specificity of skin tumor diagnosis [77,78].

Although it is a very widespread and relatively inexpensive technology dermoscopy has some limitations. The sensitivity and specificity of the diagnosis vary greatly according to the experience of the clinician and consequently a long training period is necessary [18]. However, even after short term training, dermoscopy has been shown to significantly improve accuracy in the detection of suspicious lesions in primary care [79].

New digital dermatoscopes will integrate additional capabilities in terms of magnification (i.e., super high (×400)), multiple light sources (UV, fluorescence and multispectral) and AI tools [19,80,81,82,83,84,85]. 

### 2.3. Confocal Microscopy

Confocal microscopy allows the capture of real-time cellular-resolution images of skin lesions within a few seconds, parallel to the skin surface and at different depths from the stratum corneum to the superficial dermis [20]. Two types of confocal microscopy exist: reflectance (RCM) and fluorescent confocal microscopy (FCM).

RCM uses a near-infrared power laser which emits monochromatic coherent light to scan a focal point in the skin [20,86]. The subsequent reflected light from the tissue, passing through a gating pinhole, arrives at the detector which reconstructs a grayscale image based on the relative refractive indices of the tissue elements. Melanin and keratin, which have the highest refractive index, appear brighter than surrounding tissue elements [21]. 

The basic RCM image displays a horizontal (en face) field of view of 0.5 mm × 0.5 mm but larger mosaic images up to an area of 8 mm × 8 mm (Vivablock) can be obtained. The clinician can control probe movement in the horizontal and vertical plane up to a depth of 250–300 µm. 

Two RCM devices are used in clinical practice: Vivascope 1500 (VivaScope GmbH, Munich, Germany) is the most utilized for the diagnosis of the majority of skin tumors of the body, and the handheld Vivascope 3000 (VivaScope GmbH, Munich, Germany) which is used for lesions of convex surfaces, particularly the face [87].

The main RCM clinical application is the evaluation of equivocal skin lesions with a low-to-moderate pretest probability of malignancy after dermoscopy assessment (Figure 7 and Figure 8) [20,32]. In a recent prospective multicenter randomized trial, adjunctive use of RCM for suspect lesions reduced unnecessary excisions and assured the removal of aggressive melanomas at baseline in a real-life, clinical decision-making application in referral centers with RCM [88]. 

Several studies show that RCM can increase accuracy in diagnosing MM (sensitivity (SEN) 93%, specificity (SPE) 76%) [22], basal cell carcinoma (BCC) (SEN 97%, SPE 93%) [23] and squamous cell carcinoma (SCC) with SEN and SPE ranging from 79% to 100% [24]. Although it is not always easy to evaluate crusted lesions, such as SCC, RCM can be really useful when these lesions arise on sensitive areas of the face, such as lips^47^.

Furthermore, RCM plays an important role in the evaluation of the presurgical margin of skin tumors, especially in cosmetically sensitive areas and for certain types of skin cancer, such as lentigo melanoma (LM) and BCC, associated with a significant recurrence rate after surgery [26,27].

Another possible application of RCM is the non-invasive monitoring of the response of topical or systemic treatments in skin diseases or tumors [26]. Several studies have confirmed the role of RCM in assessing the tumor clearance in those cases where clinical and dermoscopic examinations are limited because of subclinical persistence or recurrence of tumors [28,89,90,91,92].

**Figure 9:**
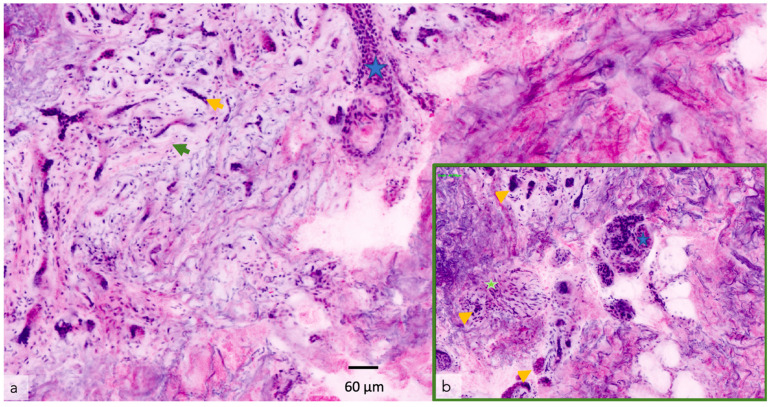
Ex vivo confocal microscopy of infiltrating basal cell carcinoma with peri neural invasion. Image captured with VIVASCOPE 2500^®^: (**a**) Yellow arrow: Tumor islands; Green arrow: Stromal reaction; Blue star: Hair follicle; (**b**) Green star: Nerve; Yellow arrow: Tumor islands; Blue star: eccrine ducts.

**Figure 10:**
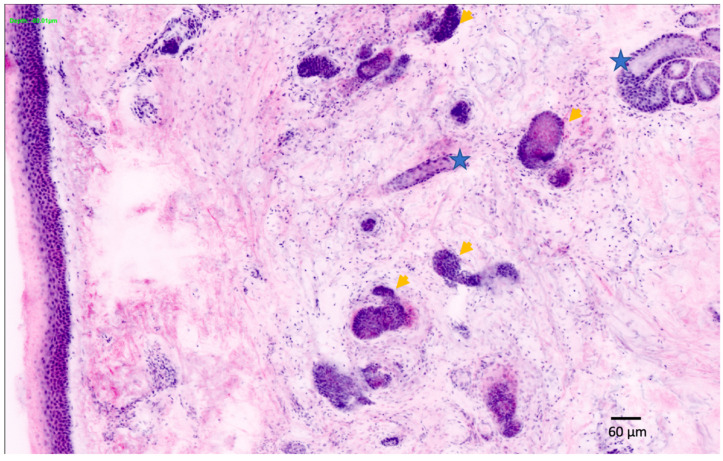
Ex vivo confocal microscopy of infiltrating peri glandular basal cell carcinoma. Image captured with VIVASCOPE 2500^®^: Yellow arrow: Tumor islands; Blue star: Eccrine glands.

In ex vivo CM, FCM uses fluorochromes, to increase the cell-to-stroma contrast [93]. Two ex vivo CM systems are currently available commercially: the Histolog Scanner (SamanTree Medical SA, Lausanne, Switzerland) and the VivaScope 2500M-G4 (VivaScope GmbH, Munich, Germany). The VivaScope 2500M-G4 uses a 785 nm/488 nm double laser which permits the acquisition of a horizontal image of 750 μm × 750 μm of the different layers of the skin with a maximum depth of scanning of 200 μm [94]. Single images are stitched together to reconstruct a mosaic image with a maximum area of 2 cm × 2 cm (Figure 9 and Figure 10).

The main application of ex vivo CM in dermatological oncology is the fast assessment of epithelial tumor margins during the excision of tumors, such as BCC and SCC of the face, associated with frequent relapses. After excision, the tissue is stained with different agents (most frequently acridine orange which targets nuclear DNA) [95] flattened [96] and then analyzed. The procedure does not damage the sample, which can subsequently be sent for histopathological examination and molecular tests.

Several studies have shown a good correlation between ex vivo CM and histopathology in the diagnosis of BCC (sensitivity 88–96.6% and specificity 89.2–99%) [29], SCC (SEN 95% and SPE 96%) [30]. However, FCM is not currently used for ex vivo assessment of melanocytic tumors due to the limited recognition of melanocytes using FCM Specific immunostainings have been tested but the time of sample processing limits the main advantage of fast diagnosis of CM and clinical validation needs to be shown [97].

Although RCM and FCM represent a revolution in non-invasive diagnostics of skin cancer, some limitations persist. The devices are expensive, and extensive training is necessary to master the procedure [31]. Furthermore, the maximum depth of penetration of the laser is about 200–300 μm, making it impossible to detect deeper lesions [32].

### 2.4. Optical Coherence Tomography

OCT is a noninvasive imaging method that can generate high-resolution en face and cross-sectional in vivo images of the skin to a maximum depth of 2 mm [98]. It is characterized by high axial resolution of 3 μm–15 μm with a good penetration depth, greater than RCM, permitting the definition of structural boundaries without reaching cellular resolution [99].

OCT images arise from the reflection of light when it passes through structures with different optical densities. In contrast to what happens with sound during an ultrasound procedure, OCT measures the depth of the different skin structures indirectly, using a technique called interferometry. This is a technique which measures the depth according to the amount of disruption to the coherence of the laser light beam as it passes through a sample compared to a reference light beam [98].

OCT has been used mainly for the diagnosis of NMSC. Some studies have shown, for dermatologists experienced with OCT imaging, a sensitivity of 86–95% and a specificity of 81–98% in diagnosing BCC [33]. Ulrich et al. demonstrated an increase in SPE in the diagnosis of non-pigmented lesions suspicious for BCC, compared to clinical-dermoscopic evaluation (Figure 1) [100].

Another possible OCT application is the differential diagnosis between actinic keratosis (AK) and Bowen’s disease and the invasive forms of SCC [101,102]. In this case, the sensitivity and specificity for experienced operators were 93.8% and 98.9% for SCC diagnosis and 81.6% and 92.6% for AK diagnosis [103]. 

Angiographic OCT (dynamic OCT), by measuring small variations in the signal intensity between two consecutive images taken in rapid succession, permits the detection of blood flow [102], achieving high-resolution two-dimensional and three-dimensional images of combined vascular structures within the skin’s structural organization [99].

Dynamic OCT has been used to evaluate vascular morphology seen in BCC [104], AK, and SCC and melanoma [105]. 

Due to the impossibility of reaching cellular resolution, the diagnosis of MM and melanocytic lesions is very challenging using OCT [41]. However, dynamic OCT has shown a certain potential in this field, permitting the detection of lesion progression via early alteration in vessel morphology from dysplastic naevus to MM [106].

**Figure 11:**
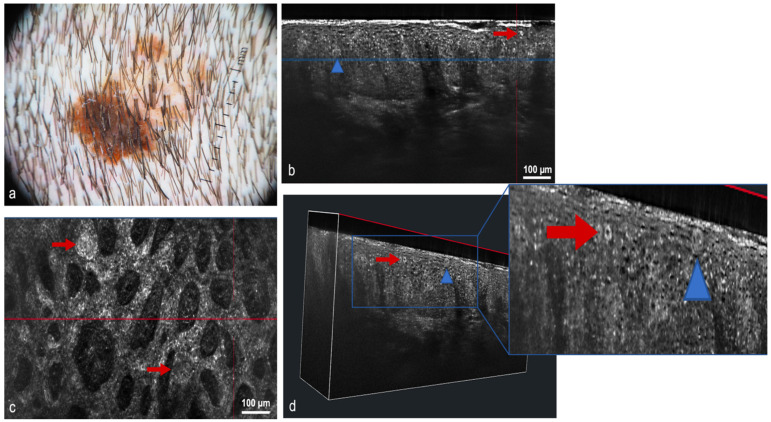
Melanoma in situ on the scalp of a 46-year-old man: (**a**) Dermoscopic image. Asymmetric lesion characterized by two areas of different color, within which it is possible to recognize some asymmetrical globules and blue-gray structures; (**b**) Vertical section. A predominantly preserved and recognizable dermoepidermal junction. In the epidermis, it is possible to recognize some atypical, bright melanocytic cells both single (red arrow) and aggregated in nests (blue triangle); (**c**) Horizontal section. Large nests of melanocytes of different sizes and shapes at the dermoepidermal junction (red arrows); (**d**) 3D reconstruction. This section makes it easier to understand the arrangements of ridges and nests. Note the presence, very high in the epidermis, of single melanocytes (red arrow) and nests (blue triangle). Detail in blue square.

In an attempt to combine the advantages of RCM (cellular resolution) and OCT (penetration depth), a new diagnostic method has been developed in recent years: line-field confocal optical coherence tomography (LC-OCT). The LC-OCT device (DAMAE Medical, Paris) combines the principle of OCT interferometry with the spatial filtering of RCM, providing three in vivo imaging modalities: histological-like vertical, RCM-like horizontal and a new unique 3-D reconstruction [35]. An axial resolution of 1.1 μm, a lateral resolution of 1.3 μm and a penetration depth of ~500 μm make LC-OCT ideal for the diagnosis and monitoring of skin tumors. Despite being a relatively new diagnostic method, some authors have already evaluated its usefulness in the diagnosis of different skin tumors including AK and SCC [36,37], and in the diagnosis and monitoring of BCC and pigmented lesions (Figure 8 and Figure 11) [38,39,40].

### 2.5. High Frequency Ultrasound

Ultrasound is a non-invasive imaging technique based on the measurement of sound wave reflections from the tissues of the body. While lower frequencies permit the exploration of deeper structures such as the internal organs, high frequency ultrasound (HFUS), with transducer frequencies of 20 MHz or more, has a lower depth of tissue penetration but produces a higher-resolution image of tissues and structures closer to the skin’s surface [107]. Indeed, frequencies of 20 MHz to 25 MHz allow visualization of skin up to the dermis while higher frequencies of 50 MHz and above visualize only the epidermis [42].

In B-mode (brightness mode), the image brightness varies according to the amplitude of the echoes: dense structures, such as keratin and collagen, generate strong echoes and are called hyperechoic; adipose tissue and lesions with a high cellularity generate weak echoes (hypoechoic), while liquids do not generate echoes and are referred to as anechoic [108].

HFUS features of the most frequent skin tumors have been described [43,44,45]. BCC appears as oval, hypoechoic lesions which usually present hyperechoic spots, with low flow arterial and venous vessels at the base of the tumor [43,44,45].

SCC images show the presence of heterogeneously hypoechoic lesions with irregular borders, and a lack of hyperechoic spots involving the deeper layers [46]. In this case, a low-flow vascular pattern is visible throughout the entire tumor, especially at the periphery.

MM appears as an oval, hypervascular, well-defined echo-poor lesion and some studies have shown a good correlation between sonographic and histologic thickness [47,48].

**Figure 12:**
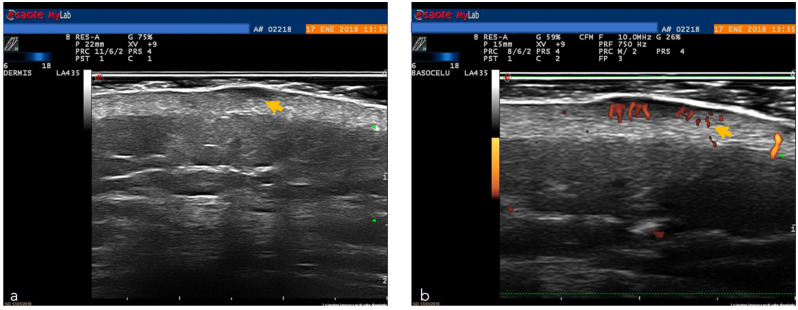
High-frequency ultrasound. A probe of 18 MHz: (**a**) Superficial basal cell carcinoma (yellow arrow); (**b**) Doppler image (yellow arrow) around the basal cell carcinoma. Image courtesy of Dr. Priscila Giavedoni.

Moreover, ultrasound can aid in the presurgical assessment of other rare tumors such as Merkel cell carcinoma (MC), dermatofibrosarcoma protuberans (DFSP), Kaposi sarcoma and primary cutaneous lymphoma (PCL) [43]. However, the reduced resolution limits the capacity to aid in the diagnosis of different subtypes of malignant skin tumors (e.g., benign vs. melanocytic lesions) [49]. HFUS ultrasound, thanks to its excellent depth of penetration, can be a useful tool in the evaluation of tumor depth and allows pre-surgical planning (Figure 2 and Figure 12) [50].

### 2.6. Raman Spectroscopy

Spectroscopy is a branch of science concerned with the spectra of electromagnetic radiation as a function of its wavelength or frequency, measured by spectrographic equipment, and other techniques, in order to obtain information concerning the structure and properties of matter [109]. The most commonly used spectroscopic techniques involve reflectance, fluorescence, or Raman scattering. 

Raman scattering is based on the vibrational modes of molecules [51]. Incoming light photons colliding with skin molecules are absorbed or scattered. Most of the scattered photons conserve their energy (elastic scattering), while a small proportion undergo a slight change in their energy when their path is modified (inelastic scattering). The energy difference between the incident and inelastically scattered photons is known as the Raman effect (or shift) and manifests as a color shift in the scattered photons which can be detected by a spectrometer [51]. Because of the difference in molecular composition between diseased tissue and normal tissue, Raman spectra can distinguish between pathological and healthy states.

In recent years, Raman spectroscopy has been used for the diagnosis of skin tumors both in an ex vivo and in vivo context. 

Earlier studies evaluating the diagnosis of NMSC and MM using a spectrometer with 1064 nm excitation achieved a sensitivity of 85% and specificity of 99% using a neural network classification model [52,53]. Unfortunately, this ex vivo technique was time-consuming and had invasiveness as its main limitation. 

For these reasons, in vivo real-time Raman systems for skin cancer diagnosis, with 785 nm excitation and reduced spectral acquisition time to less than a second, have been developed [54,55] showing 64% specificity and a sensitivity level of 90%.

Recently, the use of ultrafast laser (picosecond or femtosecond laser) for excitation allowed the development of new more advanced Raman techniques such as coherent anti-Stokes Raman scattering (CARS) and stimulated Raman scattering imaging (SRS) [56,57]. However, these methods are more expensive and there is a lack of studies evaluating their efficacy in the diagnosis of skin cancers. 

### 2.7. Electrical Impedance Spectroscopy

Electrical impedance spectroscopy (EIS) assumes that neoplastic transformation of cells alters their electrical impedance [58]. This technique uses a handheld electrode-paired probe to apply a painless electrical current both to an area of affected tissue and to healthy skin, used as reference. The probe then measures the resulting current from the tissue so that an algorithm can give a score ranging from 0 to 10 (0–3 benign, 4–10 malignant) [110].

**Figure 13:**
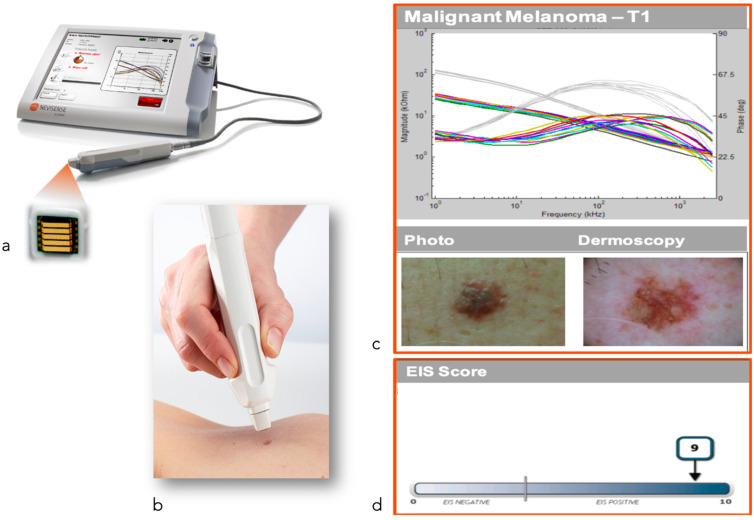
Electrical impedance spectroscopy. Nevisense^®^: (**a**) Device; (**b**) Technique; (**c**) Impedance of the lesion; (**d**) Final score and risk for malignancy.

Malvehy et al. using Nevisense (SciBase, Stockholm, Sweden) on 1,946 skin lesions, in a multicenter prospective study, found 97% sensitivity and 34% specificity for MM and 100% SEN for NMSC [59]. The results were compared to dermoscopy in a reader study, showing the superiority of Nevisense for medium level experienced dermatologists and similar for experts. Rocha et al. showed that Nevisense could reduce the need for follow-up of suspicious lesions due to its good negative predictive values [111] However, in a recent study of atypical melanocytic lesions under digital follow-up of high-risk patients for melanoma, Chavez-Bourgeois et al. showed that Nevisense may have lower sensitivity in early small melanomas which were undetectable on clinical and dermoscopic examination in this group of patients [112] The conclusion of these results is that although Nevisense showed high sensitivity in early melanoma in the pivotal study, the clinical context and population of patients may impact the outcome, as with every diagnostic method. In the future, the algorithms used in nevisense will need to consider patient’s risk factors and clinical situations (Figure 13).

Electrical impedance spectroscopy was found to be a valuable adjunct support tool in clinical decisions for cases with suspicion for NMSC in retrospective studies. In a study done by Liebich C., 1712 suspicious lesions under dermoscopy were assessed by Nevisense and 52.5% were excised. The senstivity of Nevisense was 98.4% for non-melanoma skin cancer in this study [60]. 

### 2.8. Multispectral Imaging

Multispectral imaging systems provide precise quantification of spectral, colorimetric, and spatial features of the components of the skin [61,113]. For this reason, in recent years, different devices have been developed in an attempt to improve the detection of skin cancers, particularly MM. 

In 2005, Tomatis et al. [114] used a spectrophotometric system (SpectroShadeR, MHT, Verona, Italy) for the diagnosis of pigmented lesions. The device consisted of an illumination assembly (light source, monochromator and optical fibers coupled to a probe head) and a detection device (digital color CCD camera and a digital black and white CCD camera, SpectroShadeR, MHT, Verona, Italy). 

In this case, the mirror permitted the selection of 15 different spectral bands (30 nm bandwidth) between 483 nm and 950 nm. The images taken were stored in a computer and analyzed by dedicated software, showing that multispectral image processing provided diagnostic accuracy comparable to that of expert clinicians. Because of the higher blood content of pathological tissues compared to normal skin, Bekina et al. [115] found a different ratio between the intensities of green light (545 nm), where the hemoglobin absorption is high, and red light (660 nm), where it is low. 

Additionally, other light emission systems such as halogen lamps [116] and light-emitting diodes (LEDs) [62] have been used in an attempt to improve the detection of MM and BCC.

In 2018, Rey-Barroso et al. [113] tested the use of a multispectral imaging system based on an indium gallium arsenide (InGaAs) camera and light-emitting diodes (LEDs) for the detection of skin cancer. InGaAs cameras near-infrared (exNIR) optical imaging is possible, exploring a relatively little-known spectral region ranging from 900 nm to 1600 nm. Although there are still important limitations to the method such as the low resolution of InGaAs sensors, the difficulties in selecting the exNIR LEDs and the small field of view of the system, exNIR spectral information seems to be useful in the diagnosis of skin cancer.

Another possible future application of this technology is the development of miniaturized spectral imaging systems which could be linked to smartphones [63], allowing a beneficial quantitative, mobile skin diagnosis. 

### 2.9. Multiphoton Microscopy

Multiphoton laser scanning microscopy (MPM) is based on the simultaneous excitation of a fluorophore by two or more photons [64], obtained through the use of tunable lasers which emit pulsed packets of photons of near-infrared wavelengths. Thanks to the long wavelength of the photons, this technique permits reaching a depth up to 1000 μm [117], with very low phototoxicity and background fluorescence due to the pulsed and focused nature of the excitation laser light. For these reasons, this technique has been widely used for intravital time lapse imaging of skin tumors over the last few years. Endogenous fluorophores, such as reduced nicotinamide adenine dinucleotide (NADH), flavin adenine dinucleotide (FAD) [118], keratin, melanin [119] and collagen can be used to visualize tumoral and healthy cells with MPM. Furthermore, injectable fluorescent dyes can mark the lumen of blood vessels or innate immune cells [120], allowing the visualization of the interaction between tumoral and healthy cells and the tumor microenvironment. 

In dermatology, intravital microscopy has been used in a laboratory setting to better elucidate the mechanisms of resistance and ineffectiveness of immune and targeted therapies for MM, such as programmed cell death protein 1 (PD-1) and cytotoxic T-Lymphocyte Antigen 4 (CTLA-4) blocking antibodies [121] and BRAF and mitogen-activated protein kinase (MEK) inhibitors [65].

Furthermore, MPM has proved useful in distinguishing between normal and precancerous epithelia [122] and in combination with CARS has allowed it to provide images detailed enough to resemble a hematoxylin and eosin stain [66], making it possible to have rapid intraoperative assessment. The main limitation of multiphoton microscopy is the cost of the device and the prolonged training necessary which limits its clinical diagnostic use.

### 2.10. Multispectral Optoacoustic Tomography

Multispectral optoacoustic tomography (MSOT) uses short laser pulses to achieve thermo-elastic expansion of a dye, which can be exogenous or endogenous, such as melanin [67]. The dye expansion generates ultrasound waves which can be recorded with a microphone and converted into a high-resolution three-dimensional image. 

This technique has been proposed in research studies for the noninvasive detection of sentinel lymph nodes, showing high sensitivity but low specificity [123]. In fact, the sentinel node can be detected with high accuracy, but it is impossible to distinguish between melanophages, capsular nevi, and micrometastases of a melanoma. Given the high sensitivity of the technique, new studies will be needed to show whether, in the event of a negative test result, this technique may reduce the number of sentinel lymph node biopsies.

### 2.11. Artificial Intelligence (AI)

When talking about dermatological diagnostics, the use of machine learning and AI—the human-like intelligence exhibited by trained machines—deserves a special mention [124]. These technologies can help clinicians in the process of decision making. In the case of tumor diagnostics, it has been used in both shallow and deep AI methodologies. They consist in training computer algorithms to learn from data gathered by predefined features, using deep or shallow multilayer neural networks [125]. 

Takkidin at al. [126] found that shallow models, most frequently, were built using a support vector machine (SVM) with maximum accuracy scores of 93–99% for the diagnosis of skin cancer, using relatively small data sets. On the other hand, in the case of deep models, convolutional neural networks (CNN) were the most commonly used method for skin cancer detection, with a maximum accuracy of 94–96% using medium-size data sets [126].

Haggenmüller et al. [127] systematically analyzed the current state of research on dealing with AI-based skin cancer classification involving melanoma. The studies included three main image types: dermoscopic, clinical and histopathological whole slide images (WSIs) and the authors found that all the types investigated demonstrated an at least equivalent classification performance to CNNs and clinicians. However, limits of AI in clinical practice are the under-representation of less frequent forms of skin cancer in the training sets and limited generalization, the need to explain the results, the integration into the clinical work-flow, patient and clinician perception of AI and regulatory and legal issues. AI has a high potential to help dermatologists diagnose cancer [128]. However, the reliability of AI tools is not clear, because different data set sizes, image types, and number of diagnostic classes have been used and evaluated with different evaluation metrics [129].

Furthermore Abbasi [130] showed that even if in recent years skin self-examination has become increasingly frequent, smartphone apps which use artificial intelligence to assess skin cancer risk based on images of suspicious moles, showed variable and unreliable test accuracy. Moreover, unlike within the European Union, to date, no such apps have received US Food and Drug Administration (FDA) approval. The Federal Trade Commission withdrew two apps, MelApp and Mole Detective, from the US market and fined them for “deceptively claiming” that they “accurately analyzed melanoma risk”.

## 3. Conclusions

With the advancement of technology, dermatologists can and must deal with new diagnostic tools. Some of these instruments allow a faster and more accurate diagnosis of skin neoplasms, which is necessary to ensure adequate treatment of the patient. At the same time, they require specific, lengthy training and may increase costs to health systems if used inappropriately [131,132,133].

## Figures and Tables

**Table 1:** In vivo technologies in dermatological oncology.

Technique	Applications	Limitations	Perspectives
Clinical photography	Accurate health records, providing medico-legal documentation, evaluation of the patient’s lesions over time, pre-surgical planning, educating patients [3].	Lack of the image acquisition standardization [7]	Wider use in the documentation of skin tumors, Image standards, Integration in the EHR (electronic health record), teledermatology
Total Body Photography (TBP) ^1^	TBP provides a 2-D or 3-D reconstruction of the entire skin surface, making it possible to follow up patients with a high number of atypical melanocytic lesions in a relatively short time and increasing the accuracy of the early diagnosis of melanoma [8,9,10,11]	Lack of image acquisition standardization, high prices of some TBP systems, large amount of storage space required [7]	Better resolution and faster examinations, integration, new standards (DICOM), incorporation of AI
Dermoscopy	A cornerstone of dermatologic diagnostics. Allows the visualization of skin lesions located in the epidermis and upper dermis [12,13] increasing diagnostic accuracy and reducing the benign-to-malignant biopsy ratio [14,15,16,17]	Diagnostic accuracy varies greatly according to the experience of the clinician and consequently a training period is necessary [18]	Computer image processing and AI, super-high (400×) magnification dermoscopy [19], new devices with multiple light sources, use in teledermatology, use with smartphones
Confocal microscopy (CM) ^2^	Allows the capture of cellular-resolution images of skin lesions, parallel to the skin surface, at different depths from the stratum corneum to the superficial dermis up to a depth of 250–300 µm [20]. Reflectance confocal microscopy utilizes melanin and keratin as the main endogenous chromophores [21], increasing the diagnostic accuracy of melanoma and non-melanoma skin cancers [22,23,24], allowing the evaluation of pre-surgical skin tumor margins [25,26,27] and the non-invasive monitoring of the response of treatments [28]. Fluorescent confocal microscopy, uses fluorochromes ex vivo, to increase the cell-to-stroma contrast, allowing a fast assessment of tumor margins during surgery [29,30]	The devices are expensive [31], an extensive training period is necessary to master the procedure, limited depth of laser penetration [32], large amount of storage space required	CM/OCT/DERMOSCOPIC integrated devices, increase in image capture speed, use of new ex vivo fluorochromes to allow the staining of different types of cells (i.e., melanocytes), incorporation of AI
Optical Coherence Tomography (OCT) ^3^	OCT generates high-resolution en face and cross-sectional in vivo images of the skin to a maximum depth of 2 mm. Used mainly for the diagnosis of non-melanoma skin cancer [33]. Angiographic OCT (dynamic OCT), permits the detection of blood flow, achieving high-resolution two-dimensional and three-dimensional images of combined vascular structures within the skin’s structural organization [34]. LC-OCT ^4^ combines the principle of OCT interferometry with the spatial filtering of CM, providing three in vivo imaging modalities with cellular resolution: histological-like vertical, CM-like horizontal and a new unique 3-D reconstruction [35]. It has already shown excellent performance in the diagnosis of non-melanoma skin cancer [36,37,38,39,40]	The devices are expensive, extensive training is necessary to master the procedure, lack of cellular resolution for OCT and angiographic OCT [41]	Better contrast and optical resolution, definition of dynamic OCT diagnostic criteria, application of LC-OCT to all fields of dermatological oncology, incorporation of AI
Ultrasound	Non-invasive imaging technique based on the measurement of sound wave reflections from the tissues of the body [42]. Thanks to multiple frequencies which allow different depths of penetration, this can be a useful tool in the diagnosis of skin tumors, evaluating tumor depth and allowing pre-surgical planning [43,44,45,46,47,48,49]	Low image resolution, operator-dependent examination [50] extensive training is necessary	Higher ultrasound frequencies with better resolution of skin tumors, introduction of new devices with reduced costs for the use in practice in dermatological oncology.
RAMAN Spectroscopy	Measures the different Raman spectra, due to different molecular composition, between pathological and healthy tissue [51]. It has been used for the diagnosis of skin tumors, both in an ex vivo and in vivo context, with promising results in terms of sensitivity [52,53,54,55]	Elevated cost and limited clinical use	Development of less expensive and faster devices. New studies evaluating the diagnostic performance of coherent anti-Stokes Raman scattering and stimulated Raman scattering [56,57] are needed
Electrical impedance spectroscopy	Electrical impedance spectroscopy assumes that neoplastic transformation of cells alters their electrical impedance [58]	High processing speed and sensitivity for the diagnosis of both melanoma and non-melanoma skin cancers but limited specificity [59], only validated in prospective studies for melanocytic tumors	Role as an additive diagnostic tool, assessing resection margins, evaluating the success of a specific therapeutic regime in addition to classical dermoscopy.
Multispectral imaging	Provides precise quantification of spectral, colorimetric, and spatial features of the components of the skin using different light emission systems such as halogen lamps and light-emitting diodes [60,61]. The use of a multispectral imaging system based on an indium gallium arsenide camera makes near-infrared optical imaging possible.	Low resolution of indium gallium arsenide camera sensors, difficulties in selecting the near-infrared light-emitting diodes and small field of view of the system, long acquisition time. Lack of clinical validation	Development of miniaturized spectral imaging systems which could be linked to smartphones, allowing a promising quantitative, mobile skin diagnosis [62], improving speed of acquisition. Clinical validation.
Multiphoton microscopy (MPM) ^5^	Based on the simultaneous excitation of a fluorophore by pulsed packets of photons of near-infrared wavelengths [63,64]. It has mainly been used, in a laboratory setting, to elucidate the mechanisms of the resistance of melanoma to immune and targeted therapy. However, has also proved useful in distinguishing between normal and precancerous epithelia [65]	Mainly used for research purposes. Lengthy time of acquisition	Use in combination with other techniques, such as coherent anti-Stokes Raman scattering, to reach rapid intraoperative assessment
Multispectral optoacoustic tomography (MSOT) ^6^	Measures the ultrasound wave generated by the expansion of a dye exposed to short laser pulses [66]. It has been used in research studies for the noninvasive detection of sentinel lymph nodes [67]	Very low specificity [67]	New studies are needed

^1^ Total-body photography, ^2^ Confocal microscopy, ^3^ Optical coherence tomography, ^4^ Line-field confocal optical coherence tomography, ^5^ Multiphoton microscopy, ^6^ Multispectral optoacoustic tomography.
